# Massive parallel-sequencing-based hydroxyl radical probing of RNA accessibility

**DOI:** 10.1093/nar/gku167

**Published:** 2014-02-24

**Authors:** Lukasz Jan Kielpinski, Jeppe Vinther

**Affiliations:** Department of Biology, University of Copenhagen, Ole Maaløes Vej 5, DK-2200 Copenhagen N, Denmark

## Abstract

Hydroxyl Radical Footprinting (HRF) is a tried-and-tested method for analysis of the tertiary structure of RNA and for identification of protein footprints on RNA. The hydroxyl radical reaction breaks accessible parts of the RNA backbone, thereby allowing ribose accessibility to be determined by detection of reverse transcriptase termination sites. Current methods for HRF rely on reverse transcription of a single primer and detection by fluorescent fragments by capillary electrophoresis. Here, we describe an accurate and efficient massive parallel-sequencing-based method for probing RNA accessibility with hydroxyl radicals, called HRF-Seq. Using random priming and a novel barcoding scheme, we show that HRF-Seq dramatically increases the throughput of HRF experiments and facilitates the parallel analysis of multiple RNAs or experimental conditions. Moreover, we demonstrate that HRF-Seq data for the *Escherichia coli* 16S rRNA correlates well with the ribose accessible surface area as determined by X-ray crystallography and have a resolution that readily allows the difference in accessibility caused by exposure of one side of RNA helices to be observed.

## INTRODUCTION

It is becoming clear that many RNA molecules from living cells and viruses have functions that do not depend on being translated, but rather on adopting intricate structures and binding to proteins (1,2). This is true not only for well-characterized non-coding RNAs such as ribosomal, transfer, small nucleolar RNAs and viral RNA genomes, but also for more recently discovered non-coding RNA families, such as long non-coding RNAs and microRNAs. For many of the novel non-coding RNAs that have been discovered during the past decade, the function remains unknown and even for some of those that have been functionally characterized, details of the mechanism of action are lacking. In many cases, knowledge of the tertiary structure of these RNA molecules will be necessary to identify and understand their functions. Thus, there is a clear need for structure-probing methods that can deal with the increasing number of known RNA molecules in cells. Computational methods for prediction of tertiary RNA structure are improving (3), but they still demand large computational resources, cannot be used with long RNAs and have large root mean square deviations from the experimental structures (4). Moreover, experimental methods, such as X-ray crystallography and NMR, are especially challenging for long or flexible RNA molecules (4).

As an attractive alternative, the RNA backbone solvent accessibility can be mapped by hydroxyl radical footprinting (HRF) (5–7). The hydroxyl radical reacts with hydrogen atoms on the ribose C4′ and C5′ positions in parts of an RNA molecule exposed to the solvent, leading to RNA cleavage (8). The cleavage pattern can be visualized by electrophoresis of cDNA fragments produced by reverse transcription (6). Hydroxyl radicals can be conveniently produced in solution through the Fenton reaction between Fe(II)–EDTA and hydrogen peroxide (5) or inside cells using a synchrotron X-ray beam (9). HRF can therefore be applied to many different experimental conditions and allows changes in the tertiary structure or accessibility of the RNA to be determined by comparison of the abundance of fragments produced during reverse transcription. This type of comparison is relatively insensitive to the background produced by non-specific termination of reverse transcriptase and has successfully been used to identify the changes occurring during the folding of the RNA (10) and the binding of ligands to riboswitches (11) or to map protein-binding sites on RNA (also called footprinting) (9,12). Alternatively, HRF data for RNA molecules can be compared to a non-hydroxyl radical treated control to normalize for background termination of reverse transcription and in this way produce a direct measure of the accessibility of the analyzed RNA molecule (6). Recently, it was demonstrated that such normalized HRF data anti-correlates with the number of through-space ribose neighbors, which is a measure that can be used to bias discrete molecular dynamics simulations of RNA tertiary structure prediction. Importantly, addition of the experimental data led to significant improvements in the accuracy of the predicted structures (13).

Historically, HRF data have been obtained with radioactive labeling of the reverse transcription primer, gel electrophoresis and phosphor imaging, but the current use of fluorescently labeled primers, capillary electrophoresis and automated data analysis have significantly improved the throughput of HRF experiments (14,15). Nevertheless, the capillary methods still deal with a single RNA at a time and typically provide data for only 3–400 nt in a single experiment. Thus, the throughput of HRF could be dramatically improved if its readout could be adapted to using modern massive parallel sequencing technology. This has recently been shown to be possible for SHAPE probing of RNA secondary structure allowing hundreds of *in vitro* transcribed RNA molecules to be analyzed in parallel using a single primer (16). Here, we use massive parallel sequencing together with random priming of reverse transcription and a novel barcoding and normalization scheme to dramatically improve the throughput of HRF experiments. The method allows the probing of purified RNAs and facilitates the parallel analysis of multiple RNAs or experimental conditions. Importantly, we demonstrate that HRF-Seq data correlates well with the ribose accessible surface area as determined by X-ray crystallography. The data have a resolution that readily allows the difference in accessibility caused by exposure of one side of RNA helices to be observed, suggesting that HRF-Seq can be applied in many different settings to gain insight into the functional relevance of tertiary RNA structures.

## MATERIALS AND METHODS

### Ribosome preparation

Ribosomes were purified from the *Escherichia coli* MRE600 strain (gift of Birte Vester, University of Southern Denmark) as previously described (17). Briefly, bacteria were grown in LB medium until OD_600_ was approximately 0.7, transferred to 4°C for 15 min to slowly cool down, pelleted and stored frozen. Of the pellet, 1.25 g was resuspended in 3.125 ml buffer A (20 mM Tris–HCl pH 7 at 22°C, 10.5 mM MgOAc, 100 mM NH_4_Cl, 0.5 mM EDTA and 3 mM 2-mercaptoethanol) and lyzed twice with a French press at 1000 psi. To 2.5 ml of lysate, 125 µl DNase I (Fermentas) was added followed by 20 min incubation on ice. The DNase treated lysate was centrifuged at 30 000 g for 45 min and 1 ml of supernatant was transferred onto 1 ml of 1.1 M sucrose made in buffer B (as buffer A, but with 0.5 M NH_4_Cl) and centrifuged for 15 h at 100 000 g at 4°C. The pellet was washed with buffer A and resuspended in 5 ml of buffer C (10 mM Tris–HCl pH 7, 10.5 mM MgOAc, 500 mM NH_4_Cl, 0.5 mM EDTA and 7 mM 2-mercaptoethanol) followed by 16 h centrifugation at 100 000 g at 4°C. The pellet was washed and dissolved in buffer EH (10 mM HEPES–Na pH 7.2, 10 mM MgOAc, 60 mM NH_4_Cl, 3 mM 2-mercaptoethanol). Ribosomes were precipitated by addition of 81.25 µl ethanol to 125 µl ribosomes followed by incubation 30 min at –80°C and centrifugation at 16 000 g for 15 min. The supernatant was removed and the pellet was dissolved in buffer EH lacking 2-mercaptoethanol. Just before probing, ribosomes were diluted to 10 ng/µl (NanoDrop) and incubated 5 min at 37°C.

### RNase P specificity domain preparation

A plasmid containing the sequence of the RNase P specificity domain with a structure cassette as previously described (16) was ordered as a gene synthesis from Eurofins MWG Operon. The plasmid was linearized with BsaI-HF™ restriction enzyme (New England Biolabs) and used as a template for an *in vitro* transcription reaction with T7 RNA polymerase, 0.7 mM rNTP, 6 mM MgCl2, 1 mM spermidine, 5 mM DTT and 40 mM Tris–HCl pH 8. The reaction was incubated for 90 min at 37°C, ethanol precipitated, centrifuged and resolved on a 5% polyacrylamide, 7M Urea, 1x TBE gel. The RNA product was located with UV shadowing and the band was cut out and eluted from the gel overnight in a buffer containing 250 mM NaAc and 1 mM EDTA in the presence of half of the volume of phenol. The water phase was chloroform extracted and ethanol precipitated, followed by centrifugation and resuspension in water. RNA was folded before probing as previously described (18) with modifications. Briefly, 5.5 ng/ul RNA in 140 mM KCl and 20 mM Tris–HCl was incubated for 1 min at 90°C and transferred to 37°C. After 15 min MgCl_2_ was added to the final concentration of 2.5 mM (KCl and Tris–HCl concentrations kept constant) and the mixture was incubated for 5 min at 37°C.

### Hydroxyl radical probing

Probing was performed according to the peroxidative Fenton chemistry protocol as previously described (19). Briefly, three droplets, 2 µl each, with 5 mM ferrous ammonium sulfate–EDTA, 50 mM sodium ascorbate and 1.5% H_2_O_2_ were placed on the inside walls of a tube containing 100 µl of prepared substrates (ribosomes or RNase P). The tubes were vigorously vortexed to mix the reagents and after 60-s reactions were stopped by adding 318 µl ice-cold ethanol and 10 ug of glycogen. The samples were incubated –80°C for 30 min, centrifuged and resuspended in 12.5 µl H_2_O. Control reactions were performed in parallel, but with addition of 6 µl H_2_O instead of the three aforementioned droplets.

### Sequencing library preparation

Sequencing libraries were prepared as previously described (20) with modifications. The sequences of the primers used in this study are listed in Supplementary Table S1. Briefly, 1 µl of primer (10 µM of RT_random_primer for ribosomes, 1.7 µM RT_structure_cassette for RNase P probing) was added to 5 µl of probed RNA, followed by incubation 5 min at 65°C and transfer to ice. 14 µl of a master mix was added to each reaction to obtain final concentrations of 50 mM HEPES pH 8.3, 75 mM KCl, 3 mM MgCl_2_, 0.5 mM dNTP, 0.67 M sorbitol, 0.13 M trehalose and 10 U/µl of PrimeScript Reverse Transcriptase. The ribosome probing reactions were incubated for 30 sec at 25°C, 30 min at 42°C, 10 min at 50°C, 10 min at 56°C, 10 min at 60°C and placed on ice. The RNase P probing reactions were reverse transcribed using the same thermal conditions as used for the ribosome reaction, but without the incubation at 25°C. The cDNA was recovered with RNAClean XP as described (20) (ribosomes) or ethanol precipitation (RNase P) and resuspended in 25 µl 5 mM Na–citrate pH 6. The cDNAs were diluted 200 times in H_2_O and 3 µl were mixed with 7 µl of a ligation master mix (prepared by mixing 1 volume of CircLigaseTM 10x buffer, 0.5 volume of 1 mM ATP, 50 mM MnCl_2_, CircLigaseTM enzyme, 100 µM LIGATION_ADAPTER_RB oligonucleotide and 2 volumes of 50% PEG 6000 and 5 M betaine). The ligation reaction was incubated for 2 h at 60°C, 1 hour at 68°C and 10 min at 80°C and purified with Ampure XP beads as described (20) and eluted in 16 µl H_2_O. Of 10 µM PCR_REVERSE_INDEX primer and 14 µl of PCR master mix (1.2 volume of 10 µM PCR_forward primer, 4 volumes of Phusion 5x HF buffer, 1.6 volume of 2.5 mM dNTPs, 6.8 volume of H_2_O, 0.4 volume of Phusion polymerase), 1 µl were added to 5 µl of the ligated cDNA. The reactions were incubated using the following temperature profile: (3 min, 98°C)x1, (80 s, 98°C; 15 s, 64°C; 30 s, 72°C)x4, (80 s, 98°C; 45 s, 72°C)x20, (5 min, 72°C)x1, purified with Ampure XP beads as described (20). The PCR reactions were pooled and size selected on an E-gel 2% SizeSelect gel to retain the products in the size range 200–600 bp, which were further concentrated on a PCR purification column (Qiagen) and finally purified on Ampure XP beads before being sequenced on an Illumina HiSeq system with the 2X100 paired-end protocol. The raw sequencing data is available at http://people.binf.ku.dk/jvinther/data/HRF-Seq/

### Gel electrophoresis detection of RNase P hydroxyl radical probing

The RNase P RNA was prepared and probed as described above for the sequencing-based detection. After probing, the RNA was mixed with radioactively labelled (T4 polynucleotide kinase and ATP γ-^32^P) RT_structure_cassette oligonucleotide, incubated at 65°C for 5 min and placed on ice. Of the reverse transcription master mix (2 volumes of PrimeScript 5x buffer and of H_2_O, 0.5 volumes of 10 mM dNTP), 4.5 µl was added to 5 µl of the RNA-primer mix. The sample was transferred to 42°C and after 5 min of incubation, 0.5 µl PrimeScript enzyme was added and incubation was continued for 30 min, followed by ethanol precipitation with glycogen as carrier. A sequencing ladder sample was prepared in parallel with untreated RNase P by adding 1 µl 5 mM ddATP to the reaction. The samples were dissolved in formamide loading dye (92.5% formamide, 5 mM EDTA, 0.025% bromophenol blue, 0.025% xylene cyanol), denatured (2 min, 90°C) and resolved on 40-cm long, 8% polyacrylamide, 7M Urea, 1x TBE gel at 45 W. After electrophoresis the gel was transferred onto Whatman paper, dried, exposed to image plate and scanned (Cyclone Storage Phosphor, Packard).

### Pre-processing of sequencing reads

The Cutadapt utility (21) was used to remove contaminating adapter sequences (‘-a AGATCGGAAGAGCACACGTCT’ for the first and ‘-a AGATCGGAAGAGCGTCGTGTAGGGAAAGAGTGT’ for the second read in pair) and to filter out low quality ends (‘-q 17’). Using an awk script, the 7 nt barcode was removed from the beginning of the first read and saved in separate file and the last 7 nt from the end of the second read were removed. Finally, pairs containing a read shorter than 15 nt after trimming were filtered out.

### Assembly of *E. coli* MRE600 16S rRNA sequence

The pre-processed sequence pairs were used as input for Trinity (22) to assemble the strain specific 16S rRNA sequence. Comparison of the Assembly to the sequence of chain A in 3OFA pdb structure identified five mutations (r.80a>c, r.89u>g, r.93u>c, r.183c>u and r.1498u>g).

### Mapping reads pairs to strain specific 16S rRNA sequence of RNase P specificity domain sequence

The sequence pairs were mapped to the assembly corrected 16S rRNA sequence or to the RNase P specificity domain sequence using Bowtie 2 program (23) with options ‘-N 1 -L 15 –norc -X 700’. Untemplated nucleotides, putatively added via terminal transferase activity of reverse transcriptase, were trimmed as described previously (20). For the analysis of 16S rRNA, pairs that spanned <100 nt were discarded to reduce effects of size selection.

### Estimated Unique Counts

We defined a fragment as a pair of sites (i) the termination site, which is the last reverse transcribed RNA nucleotide and (ii) the priming site, which is the first sequenced nucleotide of the second read. Relationship between the EUC (‘*n*’) and the number of observed unique barcodes (‘*k*’) was calculated using formula 1, which is an extension of a previously used method (24), but allowing different barcodes to be ligated with different probabilities (‘*P_i_*’). We calculated the frequency of the different nucleotides at each position of the barcode using the observed set of barcodes from mapped fragments having a read count within three lowest quartiles of all fragments in the given dataset (Supplementary Table S2). To estimate the *P_i_* for each barcode in each performed ligation reaction, we assumed that positions in the barcode are independent and multiplied the probabilities for all possible sequence combinations. Finally, for each experiment we sum over all possible barcodes (‘*m*’) and calculate the table of *k*(*n*) relationships, which was reversed to a *n*(*k*) table, rounded to nearest integer and used to read out the EUC (‘*n*’) for the observed (‘*k*’) for each fragment.

Formula 1:

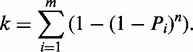



### RNase P hydroxyl radical probing gel quantification and correlation with sequencing

The scanned gel image was quantified with ImageJ (25). The signals corresponding to nucleotides 117–221 in the RNase P RNA were manually assigned to the sequence by comparison to a ddATP-sequencing reaction run in parallel. For each band the maximal value was extracted, followed by subtraction of the average signal intensity in the whole ±6-nt region to correct for unequal background intensity over the gel length. To allow optimal comparison between sequencing EUC and gel intensities, the sequencing data was not trimmed for untemplated additions to the 3′-end of the cDNA by reverse transcriptase, because we expect these shifts in signal to be present in the gel resolved fragments. For the plot in Supplementary Figure S2, we have used positions 117–186, which were chosen due to bands compression in the region before and the effect of size selection of the sequencing library in the region after.

### Number of through-space contacts in RNase P specificity domain calculation

To calculate the number of through-space ribose contacts, we have used chain B of the 1NBS pdb structure (26) with the positions 121–124 structurally aligned from chain A of the same structure. Atom locations were obtained from the PDB file and used to calculate ribose positions, defined as the mean of the C1′, C2′, C3′, C4′ and O4′ positions. Next, we used the ribose bead locations to calculate the number of ribose positions (excluding the neighbouring riboses) within distance of 14Å from a given ribose position.

### Solvent accessible surface area calculation

Solvent accessible surface area was calculated using the PyMOL get_area function with settings dot_solvent=1, dot_density=3. For the RNase P specificity domain, chain B of 1NBS structure (chain A for positions 120–125) (26) and a solvent radius of 1.4Å was used, whereas chain A of 3OFA structure in complex with 3OFC (27) and a solvent radius of 3Å was used for 16S rRNA (Supplementary Figure S1).

### Running average of ΔTCR calculation

Termination count at a given position was calculated as the sum of the EUCs of fragments terminating at the position. Effective coverage at a given position was calculated as the sum of the EUCs of the fragments terminating at or spanning the position. In addition for the ribosome analysis, fragment were only used for calculation of effective coverage for a given position, if distance between the position and the priming position was at least 100 nt. For RNase P the coverage was calculated using all fragments, but only positions 87–186 were used for the subsequent analysis. A coverage cut off was set to coverage that would provide a 90% probability that a termination count was observed given the average cleavage probability (median ΔTCR). The termination-coverage ratio (TCR) of a given position was calculated by dividing termination EUC by the effective coverage EUC. ΔTCR was calculated according to Formula 2. As a last step ΔTCR was smoothed with a moving average over a window of 3 nt and offset by 1 position upstream to reflect the fact that reverse transcription terminates before cleaved position.

Formula 2:

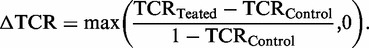



## RESULTS

### Reducing the biases in massive parallel sequencing based readout of HRF

**Figure 1.**
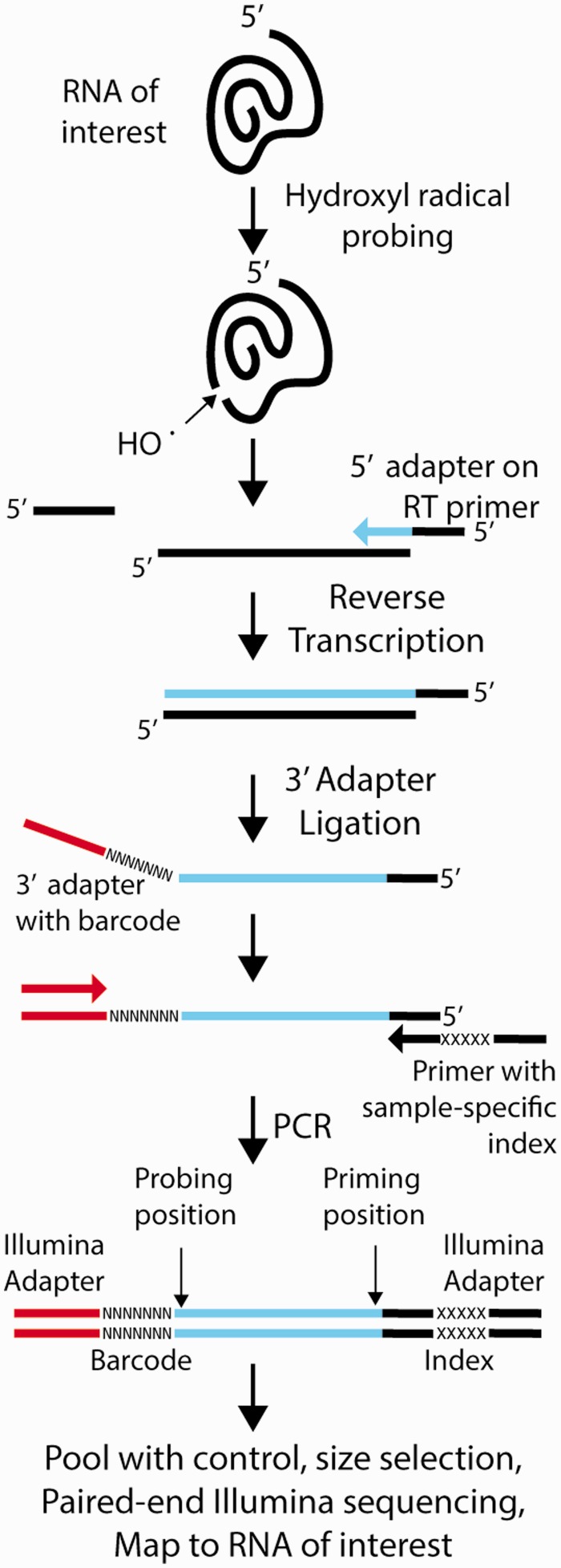
Major experimental steps of the HRF-Seq method. Following hydroxyl radical probing, primers containing a 5′ illumina adaptor overhang are extended by reverse transcriptase to positions of radical induced breaks. Adapters containing a 7-nt barcode are ligated to the 3′-ends of cDNAs, followed by PCR amplification with primers containing Illumina compatible adaptor and index sequences. After size selection, the library is sequenced with the Illumina paired-end protocol to provide information of the positions of probing and priming.

**Figure 2.**
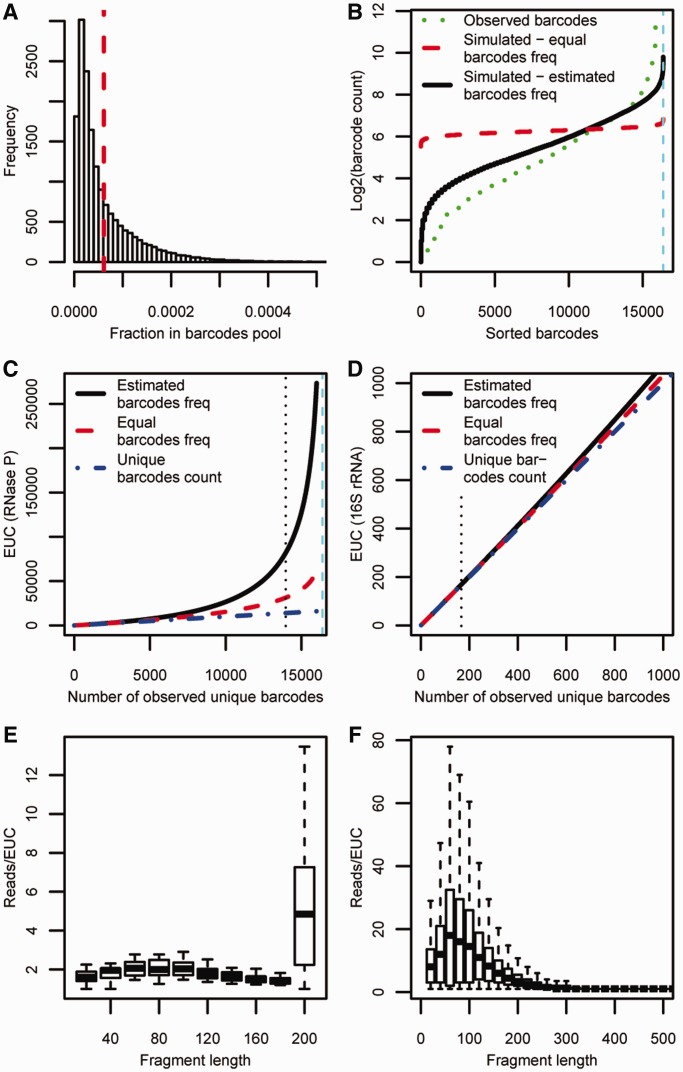
Using barcodes to estimate unique counts. (**A**) Observed barcode frequencies. Histogram showing the distribution of observed barcode frequencies in the hydroxyl radical treated RNase P experiment. The broken vertical line indicates the barcode frequency if all barcodes were present at equal frequencies. (**B**) Estimation of barcode counts. The plot compares the observed barcode counts with simulated barcode counts as estimated by assuming equal barcode frequencies or the unequal barcode frequencies as estimated by our strategy. Data is from the hydroxyl radical treated RNase P experiment. (**C**) Relationship between the number of observed unique barcodes and EUC for different types of barcode normalization strategies for the hydroxyl radical treated RNAse P experiment. The vertical line shows the highest count observed in the experiment. (**D**) Relationship between the number of observed unique barcodes and EUC for different types of barcode-normalization strategies for the hydroxyl radical-treated 16S rRNA. The vertical line shows the highest count observed in the experiment. (**E**) Length dependent bias of fragments in the probing of the RNAse P specificity domain RNA. (**F**) Length dependent bias of fragments in the probing of the 16S rRNA.

As in classic HRF, our massive parallel sequencing strategy (HRF-Seq) is based on the detection of reverse transcription termination sites, but instead of analyzing the sample on a gel or a capillary, we ligate an adaptor to the 3′-end of the cDNA and PCR amplify using primers containing adaptor and index sequences allowing massive parallel sequencing of many different conditions in a single lane on the Illumina platform (16,20) (Figure 1). After paired-end sequencing, the resulting reads can be mapped to the investigated RNA to give the precise coordinates of the priming and probing event. Compared with capillary analysis, the great advantage of using sequencing is increased throughput, but sequencing methods also introduce additional experimental biases during ligation, PCR amplification and sequencing steps (28). To reduce these biases, we introduced a 7-nt random barcode sequence in the 5′-end of the adaptor used for ligation. The barcode serves two purposes. First, it has been shown that using an adaptor pool significantly reduces ligation bias in small RNA-cloning experiments using T4 RNA ligases (29) and we expect that the same is true for the TS2126 RNA ligase (CircLigaseTM) used in this article. Second, the barcode serves as a label that is added to each fragment before introduction of PCR and sequencing biases. At low coverage the number of unique barcodes can be used directly to give the count for the specific fragment before the PCR. At high coverage, it becomes more likely that the barcodes of the same sequence are ligated to the same fragment multiple times (become saturated). Saturation occurs when the fragment count exceeds the square root of the number of barcodes and will affect the accuracy of quantification (30). By assuming that all the barcodes have equal probability of being attached to a given fragment, it is possible to correct for saturation and calculate an estimated unique count (EUC) (24). In our experiments, the ligation adaptor is prepared by standard oligonucleotide synthesis as a pool of oligonucleotides having seven degenerate positions at the 5′-end. During our analysis, we realized that the individual barcodes are present at very different frequencies in the barcode pool (Figure 2A), meaning that the observed distribution of barcodes is modeled very poorly when equal barcode frequencies in the barcode pool is assumed (Figure 2B). We therefore devised a novel strategy for estimating individual fragment counts based on the method previously implemented by Fu *et al.* (24), but taking into account that barcodes are present at different frequencies in the adaptor pool. In our strategy, the underlying barcode frequencies in the adaptor pool are estimated by determining the nucleotide frequencies observed at the seven different positions in the barcode after excluding fragments with counts in the top quartile to avoid bias from clonal amplification of specific fragments. These nucleotide frequencies are stable across our different experiments (Supplementary Table S2), suggesting that they are accurate. Assuming independence among the positions in the barcode, we then estimate the barcode frequencies by multiplication of the nucleotide frequencies. In simulation, the estimated underlying barcode frequencies produce an observed distribution of barcodes that are similar to the actual observed distribution, although the observed data still have a more extreme distribution, probably because of the presence of PCR duplicates (Figure 2B). We applied this normalization strategy to calculate EUC for HRF of a short *in vitro* transcribed RNA (specificity domain from the *Bacillus subtilis* RNase P RNA) and for HRF of a long RNA purified from cells (*E. coli* 16S ribosomal RNA), both probed with hydroxyl radicals. For the RNase P specificity domain RNA, we obtained high coverage resulting in saturation of barcodes. This is corrected using our strategy, but not using simple barcode counting or by assuming equal barcode frequencies (Figure 2C). The saturation of barcodes was not observed with the 16S rRNA, because of much lower coverage (Figure 2D). By comparing the observed fragment counts with the EUC and stratifying by fragment length, it is clear that for the RNAse P RNA, most positions have no length dependent bias (counts equals EUC) (Figure 2E). This is most likely because there is relatively little length difference between the different fragments in the PCR. For some of the RNase P positions (the longest fragments), we observe a bias, which is related to some of the barcodes containing deletions, leading to assignment of RNase P sequence as part of the barcode and subsequent reduction in the barcode complexity and underestimation of the EUC. This phenomenon will have a small, but significant effect on the quality of our data and can be avoided in the future by extending the barcode and giving it a specific signature that will allow true barcodes to be distinguished (30). For the 16S rRNA dataset, we observe a striking overrepresentation of short fragments, which is most likely caused by PCR amplification and sequencing biases (Figure 2F) and our barcode normalization strategy efficiently corrects for this bias. For both the 16S rRNA and the RNase P RNA, the EUC calculated using unequal barcode frequencies performs at least as well as the other normalization strategies when comparing with accessibility data obtained from the crystal structures (Supplementary Table S3). The superior performance of our method in determining the RNase P accessibility stems mainly from saturation of barcodes for the fragments that reach the RNA fragment terminus, leading to underestimation of signal in the other type of barcode normalization. In contrast, the 16S rRNA coverage is lower, so that a simple count of unique barcodes allows the data to be normalized for fragment length bias of PCR. Thus, our barcoding strategy corrects for fragment length bias and for the barcode saturation that can occur at high coverage, allowing the strategy to be used regardless of the level of coverage.

### HRF-Seq analysis of *in vitro* transcribed RNAse P RNA

**Figure 3.**
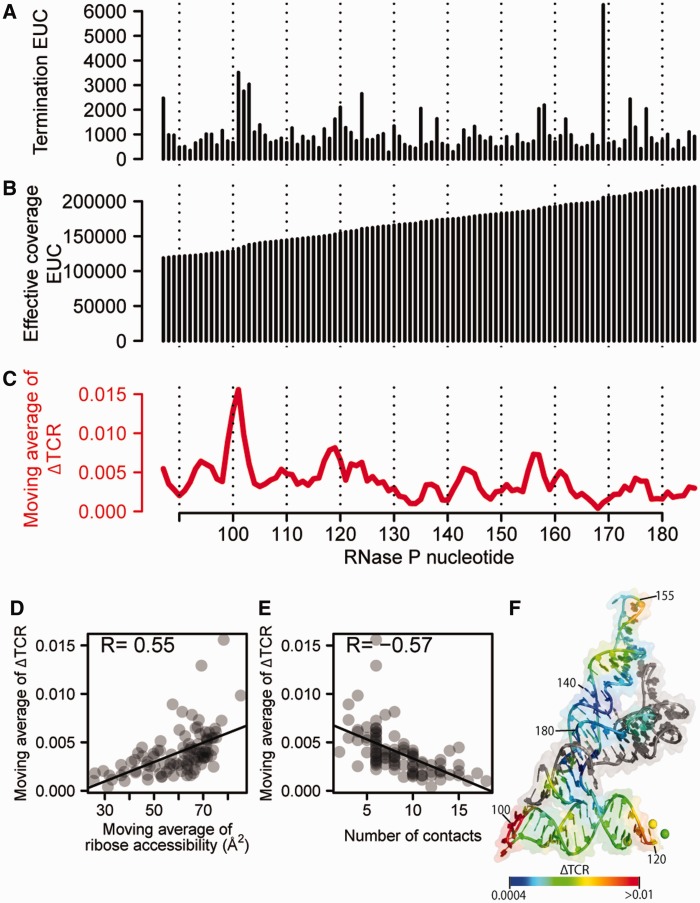
HRF-Seq analysis of RNase P RNA specificity domain. (**A**) Termination signal for HRF-treated sample calculated as the sum of EUC for fragments terminating at a given position. (**B**) Coverage for HRF treated sample. (**C**) Normalized HRF-Seq signal calculated as the 3-nt moving average of the termination coverage ratio for the HRF-treated sample with the termination coverage ratio for the control sample subtracted. (**D**) Correlation between the normalized HRF-Seq signal and a 3-nt moving average of ribose accessibility from the published crystal structure (26) using a 1.4Å probe. (**E**) Correlation between the normalized HRF-Seq signal the number of ribose through-space contacts from the published crystal structure (26). *R* values are calculated using the Pearson correlation. (**F**) Normalized HRF-Seq signal displayed on the crystal structure of the RNase P RNA specificity domain (26), gray indicates no data.

To validate our sequencing based output of HRF, we first compared the EUCs obtained for the specificity domain of *B. subtilis* RNase P RNA with the output obtained with classical gel-based HRF using identical conditions and the same primer for reverse transcription. The footprinting signals from the two methods are strongly correlated (*R* = 0.80), showing that the HRF-Seq EUC captures the same signal as classical HRF (Supplementary Figure S2). The HRF signal (Figure 3A) contains both background signal caused by spontaneous termination of the reverse transcriptase and a signal decay resulting from termination of reverse transcriptase before the probed position. To normalize for the background, we implemented a slightly modified version of the QuShape normalization method recently described by Weeks and colleagues for analysis of SHAPE data (15). In line with the QuShape method, we estimate the coverage across the RNA by summing the EUC for the fragments that reach or pass a given position (Figure 3B). The observed coverage is a measure of number of reverse transcriptases reaching a given position. This can be used to normalize the termination EUC to give a TCR, which is the fraction of reverse transcriptases that will terminate at a given position. The TCR of the treated sample is composed of probing signal and background signal, whereas the control samples’ TCR is composed of background signal only. Comparing the sum of TCR for the control and treated experiments after excluding the 5′ run off indicates that the treated RNase P sample contains 47% background signal. Assuming that background causes the same fraction of reverse transcriptases to terminate at a given position in the control and treated sample, the probing signal can be normalized for spontaneous termination of the reverse transcriptase by subtraction of the control sample TCR from the treated sample TCR to give a normalized accessibility measure ΔTCR (see Materials and methods section for full description). This is slightly different from the QuShape procedure, which assumes that the background signal in the probed sample is a scaling of the signal observed in the control sample. The median ΔTCR is a measure of the average hydroxyl radical-induced cleavage probability and for RNAse P probing it is 0.0033 (Supplementary Figure S3A and B) corresponding to 1 hydroxyl radical-induced cleavage per 300 nt and ∼34 % probability of observing a single hit on the RNA. HRF data is known to have high background signal and in some cases, barcode assignment and terminal transferase activity of reverse transcriptase can cause the signal to shift by 1 or 2 nt. In order to reduce the overall experimental noise, we therefore take advantage of the accessibility of neighboring positions being highly correlated and calculate the moving average of ΔTCR in a 3-nt window (Figure 3C). Comparing the moving average of ΔTCR with the moving average of ribose accessibility calculated from the solved crystal structure for the RNase P specificity domain RNA, we find a significant correlation (*R* = 0.55) (Figure 3D). This correlation is slightly higher than previously observed for this RNA using traditional HRF based on capillary analysis (13). Moreover, we also find that the moving average of ΔTCR anti-correlates with through-space ribose neighbors (*R* = –0.57) as calculated from the RNAse P crystal structure (Figure 2E), suggesting that HRF-Seq data can be used to inform discrete molecular dynamics simulations of RNA tertiary structure prediction (13). In the comparison with the crystal structure accessibility, we observe four positions (positions 99–102) that are clear outliers in our probing data, giving too high ΔTCR signal. This region is a loop (Figure 3F) and the discrepancy between our data and the data from the crystal structure probably reflects that this loop is more flexible and has a higher accessibility in solution.

### Random primed HRF-Seq analysis of purified 16S rRNA

**Figure 4.**
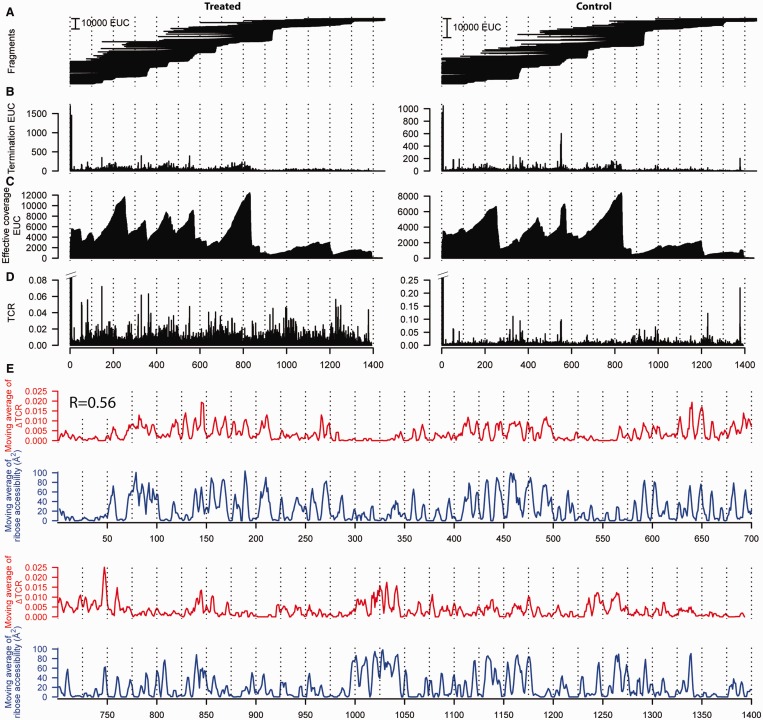
HRF-Seq analysis of *E. coli* 16S rRNA. (**A**) Sequenced fragments (EUC) from the treated (left) and control (right) sample mapped to 16S rRNA sequence. Left terminus of each fragment corresponds to the reverse transcription termination site and the right terminus to the priming site. (**B**) Sum of EUC termination signal at each position for HRF-treated and control sample. (**C**) EUC-based coverage for HRF-treated and control sample. (**D**) TCR calculated by dividing the termination signal with the coverage for the treated and control samples. (**E**) Top graph (red) shows normalized HRF-Seq signal calculated by subtracting TCR for the control sample from the TCR obtained from the treated sample and taking the 3-nt moving average. Bottom graph (blue) shows the area of ribose accessibility calculated from the crystal structure (27) as the 3-nt moving average of the accessibility to a probe with 3Å radius. *R* calculated using the Pearson correlation.

**Figure 5.**
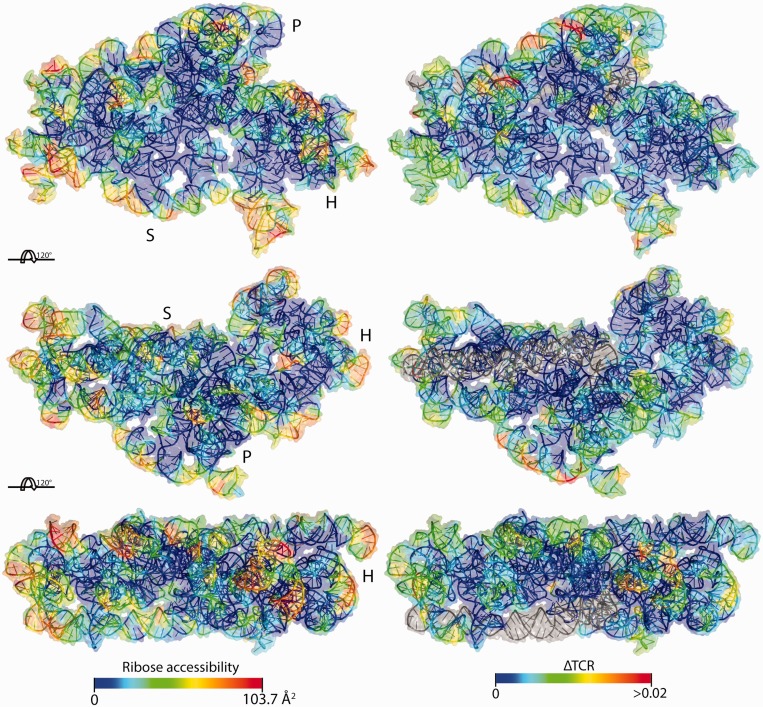
16S rRNA accessibility surface representation HRF-Seq data. (**A**) Three views of the crystal structure of the RNA part of the 16S small ribosomal subunit colored with moving average of ribose accessibility as measured from the crystal structure (27) using a 3Å probe. P, H and S indicate the platform, head and shoulder of the ribosomal subunit as named in (33). (**B**) Crystal structure of 16S small ribosomal subunit colored with the normalized HRF-Seq signal, gray indicates no data.

**Figure 6.**
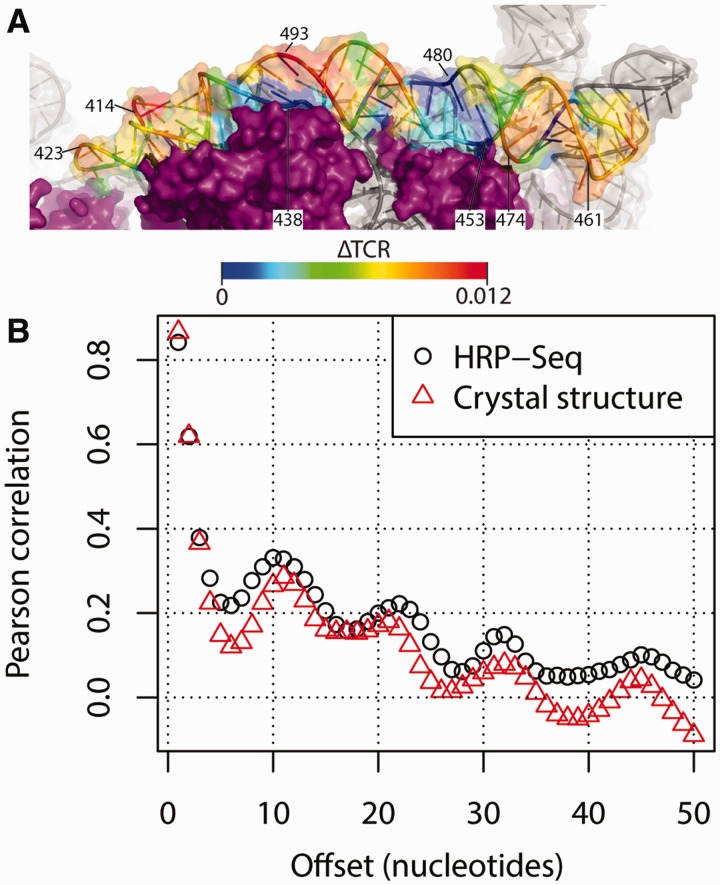
Periodicity of RNA accessibility. (**A**) Close-up of the positions 400–500 of the 16S rRNA colored with the normalized HRF-Seq signal. (**B**) Pearson correlation between HRF-Seq signal and ribose accessibility from the crystal structure for nucleotides separated by the indicated offset.

Next, we wanted to extend HRF-Seq to the analysis of long RNA molecules isolated from the cellular environment. To make our strategy general and applicable to the entire transcriptome, we used random primers for reverse transcription, rather than the single primer strategy that we used for the RNase P experiments and that were previously used for SHAPE-Seq (16). We chose the *E. coli* 16S ribosomal RNA for validation of our strategy, because of the high abundance of the ribosome and the solved crystal structure (27). Native ribosomes including ribosomal proteins were purified and used for HRF-Seq using random priming during reverse transcription to obtain signals for the entire 16S RNA molecule in a single experiment. We also obtained data for the 23S rRNA, but because of low stability during purification and high prevalence of posttranscriptional modifications that terminate reverse transcription, only parts of the 23S rRNA were covered. After mapping the reads to the 16S rRNA, we again used the barcodes present in the ligation adaptors to calculate the EUC for each observed fragment (Figure 4A). The fragments can be collapsed to give EUC for each termination position (Figure 4B). Knowing the EUC and the exact probing and priming position for each fragment, we can calculate the effective coverage at each position by taking the size selection that occurs during preparation of the sequencing library into account. In our set-up a fragment size cut-off of 100 nt ensures that the effective coverage of a position is affected only by the molecules that potentially could have been observed at the specific position given their priming site. The data for the hydroxyl radical treated sample and the control were obtained using 5.7% of an Illumina HiSeq lane. For the treated sample, 12% of 5.2 million reads mapped to 16S and provided good coverage across the large majority of the 16S rRNA (Figure 4C). Using the termination EUC and the effective coverage, we then calculated TCR for the hydroxyl treated sample and the control experiment (Figure 4D). Comparing the sum of TCR for the control and treated experiments after excluding the 5′ run off indicates that the treated sample in this case contain 86% background signal. Surprisingly, we observe a couple of positions that have very high signal in the control compared to the treated sample (most notably position 330, 551, 552 and 1378). As the only difference between the treated and control sample is the radical treatment, we speculate that these signals are the result of a nuclease activity that co-purifies with the ribosome and becomes inactivated by the radical treatment. We subtracted the control TCR from the treated TCR to give a ΔTCR value for each position. The median ΔTCR is 0.0018, which corresponds to 1 hydroxyl radical-induced cleavage per 560 nt on average (Supplementary Figure S3C and D). Finally, we applied the 3 nt window moving average to ΔTCR to give accessibility values for the 16 S *E. coli* rRNA. We find that the RNA accessibility calculated from the ribosomal crystal structure (27) as a 3 nt moving average of ribose solvent accessibility using a solvent radius of 3Å correlates with the HRF-Seq determined ΔTCR (*R* = 0.56) (Figure 4E). While the agreement between the crystal structure accessibility and the HRF-Seq data in general is quite striking, 16SrRNA positions 723 and 729 shows high signal in the HRF-Seq data, but are inaccessible in the crystal structure. In the ribosome crystal, position 723 of the 16S rRNA is bound and hidden from solvent by ribosomal protein S21 (RPS21) and RPS21 has previously been shown to crosslink to position 723 (31). Interestingly, RPS21 is known to have a fast off rate and exchange rapidly in reconstitution experiments (32) and is therefore likely to have been lost during purification, which would explain the discrepancy between our data and the crystal structure at this position. Positions 723 and 729 are located in a loop and the high HRF-Seq signal at position 729 compared to the crystal accessibility indicates that the loop changes its conformation when RPS21 is absent, thereby exposing position 729 to the solvent. In general, however, the footprints of ribosomal proteins and the large ribosomal subunit on the 16S surface are readily observed in HRF-Seq data (Figure 5). As exemplified by position 723, the resolution of the HRF-Seq accessibility signal is high. Zooming in on H16/H17, which run parallel to the long axis of the subunit and are located on a rather flat surface, it is clear that HRF-Seq allows the difference in accessibility caused by exposure of one side of RNA helices to be attained (Figure 6A). In fact, even for the entire 16S molecule, we observe a strong correlation in accessibility signal for positions separated by one or two helical turns (Figure 6B), probably because a significant fraction of 16S rRNA is helical and exposed on the surface. As expected for accessibility footprinting there is no significant difference in the HRF-Seq signal for base-paired positions compared to non-base-paired positions, but interestingly the probing signal of positions that are Watson–Crick base-paired correlates with the probing signal of positions on the opposite strand located downstream (offset by two and three bases) from the paired position (*R* = 0.41 and 0.43, respectively). This is in perfect agreement with what one would expect from the accessible surface area of riboses in helical structure with one side facing the solvent.

## DISCUSSION

We present a new method for HRF of RNA backbone accessibility using massive parallel sequencing as the readout. Our study demonstrates that this method has dramatically improved throughput compared to classical capillary-based methods and produces data that agree well with RNA ribose accessible surface areas and through-space contacts determined by the X-ray crystallography. Importantly, we show that HRF-Seq makes it possible to analyze long RNA molecules and mixtures of RNA molecules in parallel in a single tube by using random primers. To this end, we devised new strategies for reducing PCR and sequencing biases based on barcodes in the ligation adaptor and on data normalization using the probing and priming position information obtained during sequencing. Both of these strategies could be implemented for other types of sequencing based probing methods. During the final preparation of this article, *Seetin et al.* published a method to reduce the bias in probing experiments based on the detection of termination of reverse transcription also by introducing barcodes, but only for *in vitro* transcribed RNAs with a single primer (34). An important advantage of using massive parallel sequencing as readout for HRF experiments is the digital nature of the data, which makes data processing relatively easy compared to the analysis of data obtained by gel or capillary electrophoresis. Moreover, after mapping we find that a substantial fraction of the reads (∼20% on average) have mismatches in the three positions corresponding to the very 3′-end of the cDNA produced, which is indicative of untemplated nucleotides being added to the cDNA by the terminal transferase activity of the reverse transcriptase. This causes a shift of signal in the 5′ direction of the RNA, which cannot be corrected when using gel and capillary-based methods for data readout. In contrast, using massive parallel sequencing readout, we can perform a simple trimming of reads with terminal mismatches to correct the probing position for ∼75 % of cases with untemplated nucleotides added (20).

HRF is a versatile method that can be used to investigate changes in tertiary RNA structure, identify protein footprints on RNA and guide the computational prediction of tertiary RNA structure. Here, we compare a radical treated sample with a control sample to obtain an accessibility signal that could be used for computational prediction of tertiary RNA structure by calculating ΔTCR and averaging it over three positions. The averaging not only improves overall correlation because of the high accessibility correlation with neighboring position observed in the dataset (Figure 6B), but also blurs the fine details. In other types of experiments, such as typical footprinting experiments, where two probed conditions are compared, the objective will be to determine specific position that have differential accessibility in the two conditions. In such cases, it would make sense to analyze the data by comparing the coverage and termination EUCs of the two samples with the Fisher exact test or a test based on the negative binominal distribution. In this way, the coverage and termination count will be taken into account in the calculation of the significant differences between the two samples. Importantly, the use of X-rays allows HRF to be performed inside intact cells (9) and kinetic studies of RNA folding (10) to be performed. HRF-Seq should be readily applicable to such types of analysis and we therefore expect that the throughput provided by HRF-Seq will help pave the way for an increased understanding of the functional consequences of RNA tertiary structure inside cells and the dynamics of RNA folding. In particular, HRF-Seq should facilitate the probing of long RNA molecules, such as mRNAs, long ncRNAs and viral RNAs, for which tertiary structure information currently is very limited.

## SUPPLEMENTARY DATA

Supplementary Data are available at NAR Online.

## FUNDING

Danish Council for Strategic Research [Center for Computational and Applied Transcriptomics, DSF-10-092320]; Department of Biology, University of Copenhagen (PhD fellowship to L.J.K.). Funding for open access charge: The Danish Council for Strategic Research.

*Conflict of interest statement*. None declared.

## Supplementary Material

Supplementary Data
