# Supplementary material for: Massive parallel-sequencing-based hydroxyl radical probing of RNA accessibility
Source: Nucleic Acids Res. 2014 Feb 24;42(8):e70. doi: 10.1093/nar/gku167 (PMC4005689; doi:10.1093/nar/gku167)
Supplement: Supplementary Data [file supp_42_8_e70__index.html]

Massive parallel-sequencing-based hydroxyl radical probing of RNA accessibility — Supplementary Data 

# Massive parallel-sequencing-based hydroxyl radical probing of RNA accessibility

## Supplementary Data

files

**Files in this Data Supplement:**

- Supplementary Data - pdf file
- Supplementary Data - xls file
