# Supplementary material for: Massive parallel-sequencing-based hydroxyl radical probing of RNA accessibility
Source: Nucleic Acids Res. 2014 Feb 24;42(8):e70. doi: 10.1093/nar/gku167 (PMC4005689; doi:10.1093/nar/gku167)
Supplement: Supplementary Data [file supp_gku167_nar-03510-met-f-2013-File009.pdf]

Supplementary information accompanying the paper:

Massive parallel sequencing based hydroxyl radical footprinting of RNA accessibility

Lukasz Jan Kielpinski[1], Jeppe Vinther[1,*],

[1]Department of Biology, University of Copenhagen, Ole Maaløes Vej 5, DK-2200 Copenhagen N, Denmark

CONTENT

Supplementary figures 1-3

Supplementary tables 1-3

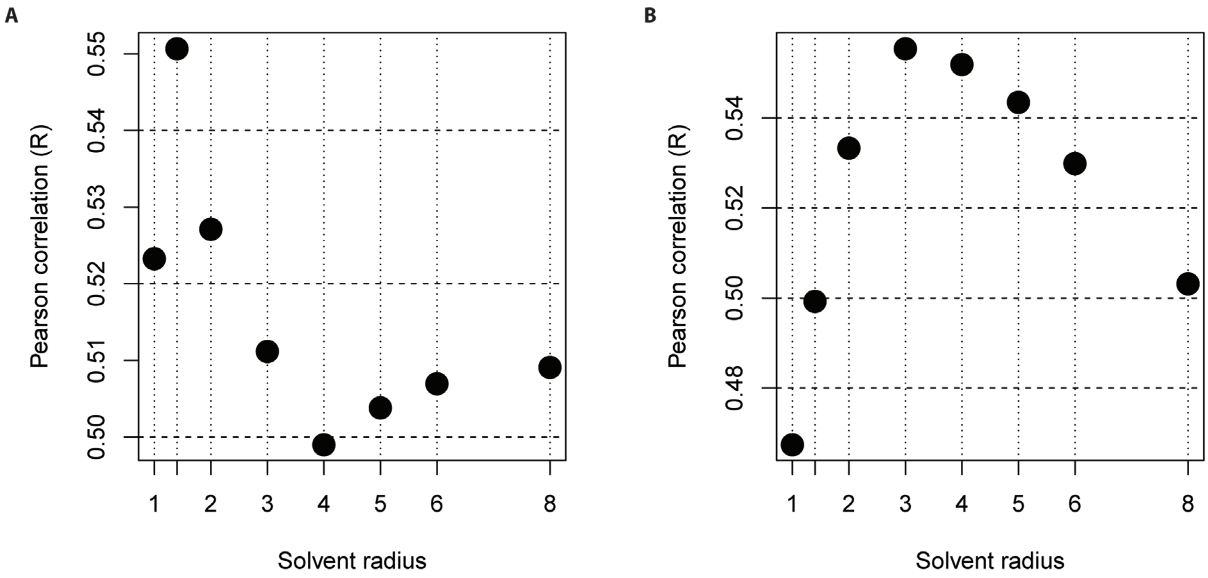

Supplementary Figure 1. Impact of solvent radii used for calculation of ribose accessible surface area on the correlation with HRF-Seq data.

A) Correlation between moving average of ΔTCR and moving average of ribose accessible surface area calculated with different solvent radii for RNase P. Highest correlation was observed for probe radii 1.4 Å. **B)** Correlation between moving average of ΔTCR and moving average of ribose accessible surface area calculated with different solvent radii for 16S rRNA. Highest correlation was observed for probe radii 3 Å.

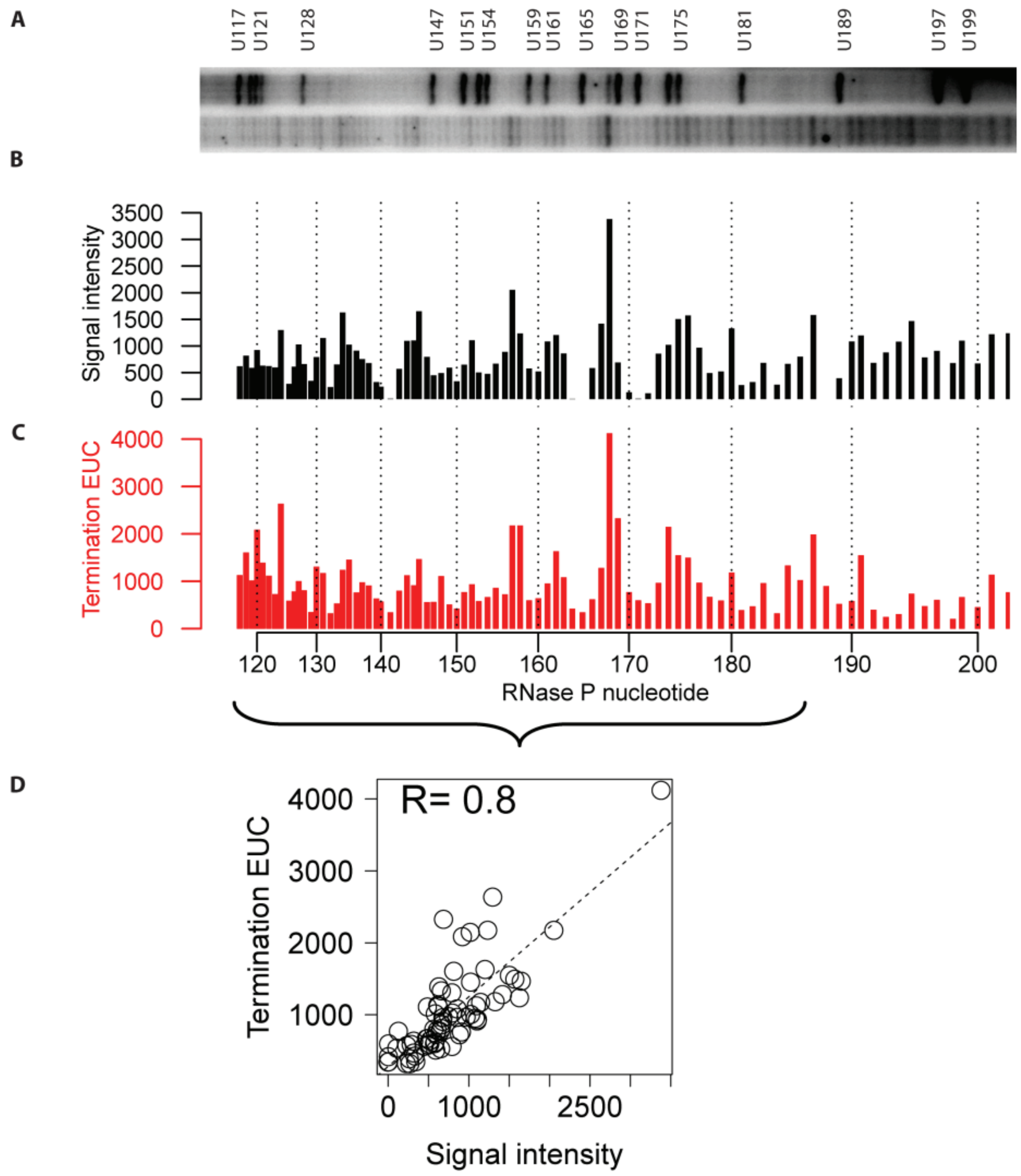

Supplementary Figure 2. Comparison of classical hydroxyl radical probing with HRF-Seq. **A)** Autoradiogram of gel electrophoresis of RNase P hydroxyl radical probing (lower lane) with the ddATP sequencing as size marker (upper lane). **B)** Quantification of gel shown on plot A. **C)** Termination EUC as obtained in RNase P-treated sequencing experiment. **D)** Correlation plot between signal intensity and termination EUC. The shown R value is the Pearson correlation.

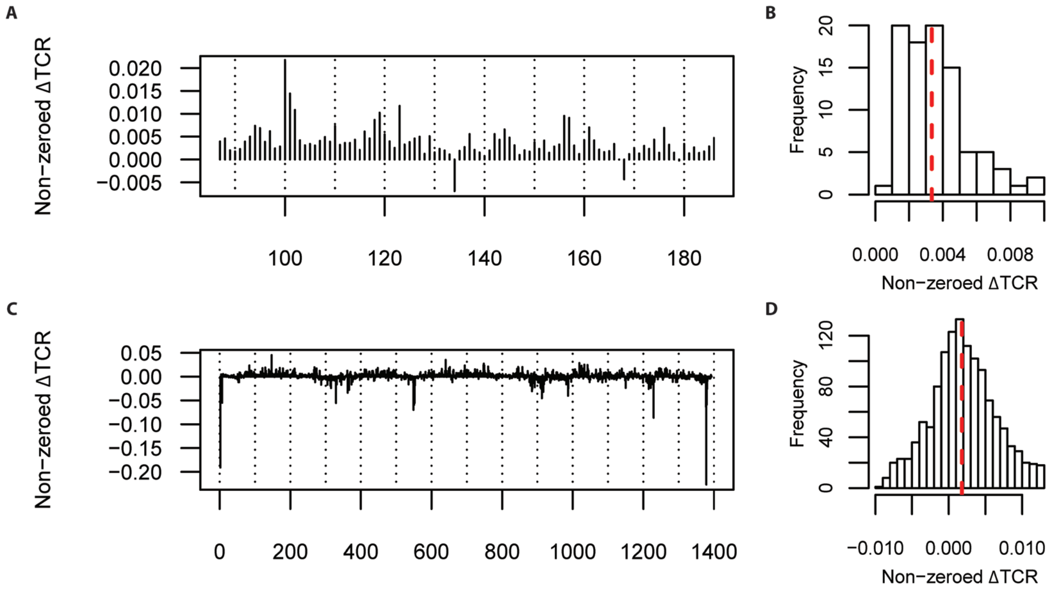

Supplementary Figure 3. ΔTCR values before averaging and zeroing.

A) Barplot of non-zeroed ΔTCR for the footprinting of the RNAseP RNA. **B)** Distribution (excluding 5% top and 5% bottom values) of non-zeroed ΔTCR for the footprinting of the RNAseP. Dashed, vertical red lines represent the median ΔTCR. **C)** Barplot of non-zeroed ΔTCR for the footprinting of the 16S rRNA. **D)** Distribution (excluding 5% top and 5% bottom values) of non-zeroed ΔTCR for the footprinting of the 16S rRNA. Dashed, vertical red lines represent the median ΔTCR.

Oligonucleotide name	Oligonucleotide sequence (5’ to 3’)
RT_random_primer	AGACGTGTGCTCTTCCGATCTNNNNNNNNS
RT_structure_cassette	AGACGTGTGCTCTTCCGATCTGAACCGGACCGAAGCCCG
LIGATION_ADAPTER_RB	PHO-NNNNNNNAGATCGGAAGAGCGTCGTGTAGGGAAAGAGTGT-3NHC3
PCR_forward	AATGATACGGCGACCACCGAGATCTACACTCTTTCCCTACACGACGCT
PCR_REVERSE_INDEX.14_AGTTCC	CAAGCAGAAGACGGCATACGAGATGGAACTGTGACTGGAGTTCAGACGTGTGCTCTTCCGATCT
PCR_REVERSE_INDEX.16_CCGTCC	CAAGCAGAAGACGGCATACGAGATGGACGGGTGACTGGAGTTCAGACGTGTGCTCTTCCGATCT
PCR_REVERSE_INDEX.22_CGTACG	CAAGCAGAAGACGGCATACGAGATCGTACGGTGACTGGAGTTCAGACGTGTGCTCTTCCGATCT
PCR_REVERSE_INDEX.24_GGTAGC	CAAGCAGAAGACGGCATACGAGATGCTACCGTGACTGGAGTTCAGACGTGTGCTCTTCCGATCT

Oligonucleotide sequences © 2007-2009 Illumina, Inc. All rights reserved.

Supplementary Table 1. Oligonucleotides used in the study

Sample	Sequenced nucleotide	Sequenced position						
		1	2	3	4	5	6	7
16S rRNA, Treated	A	0.26	0.26	0.24	0.27	0.3	0.42	0.08
	C	0.3	0.33	0.33	0.31	0.28	0.28	0.55
	G	0.19	0.17	0.19	0.19	0.17	0.1	0.09
	T	0.25	0.24	0.24	0.23	0.24	0.19	0.28
16S rRNA, Control	A	0.25	0.25	0.24	0.28	0.31	0.43	0.08
	C	0.3	0.33	0.32	0.31	0.28	0.27	0.58
	G	0.2	0.18	0.19	0.19	0.17	0.1	0.09
	T	0.25	0.24	0.25	0.22	0.24	0.2	0.26
RNase P, Treated	A	0.27	0.26	0.25	0.27	0.3	0.41	0.09
	C	0.29	0.33	0.33	0.31	0.29	0.28	0.52
	G	0.2	0.17	0.18	0.19	0.17	0.1	0.09
	T	0.24	0.24	0.24	0.23	0.24	0.2	0.3
RNase P, Control	A	0.26	0.26	0.25	0.27	0.3	0.42	0.09
	C	0.3	0.33	0.32	0.3	0.29	0.28	0.54
	G	0.2	0.17	0.19	0.2	0.17	0.1	0.09
	T	0.24	0.24	0.24	0.23	0.25	0.2	0.28

Supplementary Table 2. Nucleotide frequencies at each barcode position for each sample used to calculate the barcode ligation probabilities.

Sample	Counting reads	Counting unique barcodes	EUC equal barcode frequencies	EUC estimated barcode frequencies
RNase P	0.45	0.50	0.53	0.55
16S rRNA	0.49	0.56	0.56	0.56

Supplementary Table 3. Pearson correlation between HRF-Seq signal and ribose accessibility for different methods of processing the sequencing data
